# Sun Exposure Public Health Directives

**DOI:** 10.3390/ijerph15122794

**Published:** 2018-12-10

**Authors:** David G. Hoel, Frank R. de Gruijl

**Affiliations:** 1 Department of Public Health Sciences, Medical University of South Carolina, Charleston, SC 29425, USA; 2 Department of Dermatology, Leiden University Medical Center, 2333 ZA Leiden, The Netherlands; f.r.de_gruijl@lumc.nl

**Keywords:** UV radiation, vitamin D, nitric oxide, melanoma, cancer, cardiovascular

## Abstract

There have been many public health recommendations for avoiding UV radiation exposures. This is primarily due to concerns about skin cancer and especially melanoma, the most serious type of skin cancer. However, UV radiation is also known as the primary source of vitamin D and other compounds needed for good health. This brief commentary lists several of the many important recent studies of adverse health effects associated with low sun exposure, including some specific cancers, multiple sclerosis, diabetes, cardiovascular disease, autism, Alzheimer’s disease, and age-related macular degeneration. Our conclusion is that non-burning UV exposure is a health benefit and—in moderation—should be recommended as such.

## 1. Commentary

There is currently considerable misinformation and confusion in the public health sector regarding the effects of sun exposure on human health. The prevailing public health message is, and has been for the past several decades, that “over exposure” (undefined) to the sun causes skin cancer, including melanoma, and health benefits of sun exposure are limited to bone health. Since “over exposure” is not defined, the public is led to believe that sun exposure should be avoided and that avoidance of sun exposure is risk-free. The prevailing public health message also incorrectly states that vitamin D supplements are an adequate substitute for sun exposure. This public health message is potentially causing significant harm to public health and should be changed immediately. Examples of this public health message are currently extant in the United States [1,2,3], the United Kingdom [4], Australia [5], and New Zealand [6].

An examination of the current state of the scientific research shows that (i) severe sunburns are linked to increased risk of melanoma but non-burning sun exposure is linked to reduced risk of melanoma [7,8,9]; (ii) squamous cell carcinoma of the skin (SCC) is linked to outdoor professions and correspondingly a great deal of cumulative lifetime sun exposure (20,000+ hours for northern Europeans [10] and 50,000+ hours for southern Europeans [11,12]); (iii) the linkage with UV exposure is less clear for basal cell carcinoma of the skin (BCC) than for SCC, but BCC appears to be linked mostly to sunburns rather than to cumulative lifetime sun exposure [12,13]; and (iv) the health risks of sun avoidance are indicated to be significant. One recent study estimated that approximately 12% of U.S. deaths (340,000 persons per year) may be linked to inadequate sun exposure [14], and another all-cause mortality study found that avoidance of sun exposure is a risk factor for death of a similar magnitude as smoking [15].

It is known that UV-A from sun exposure increases circulating nitric oxide, decreasing hypertension and cardiovascular disease [16]. It has been estimated that 25(OH)D concentrations (a marker for sun exposure) of less than 12 ng/mL vs. more than 33 ng/mL are correlated to 100% increased risk of colorectal cancer [17]; 25(OH)D concentrations of 19.8 ng/mL vs. 27 ng/mL are correlated with a 37% increased risk of breast cancer [18]; 25(OH)D concentrations of less than 20 ng/mL vs. more than 60 ng/mL are correlated with a 400% increased risk of breast cancer [19]; 25(OH)D concentrations of 14 ng/mL vs. 32 ng/mL are correlated with a 79% increased risk of death from breast cancer in breast cancer patients [20]. For non-cancer effects, a 25(OH)D concentration of less than 5 ng/mL vs. more than 20 ng/mL is correlated with a 35% increased risk of type 2 diabetes [21]. Serum 25(OH)D concentrations of less than 20 ng/mL are correlated with a 64% increased risk of metabolic syndrome in the elderly [22]. Serum 25(OH)D concentrations of below 10 ng/mL vs. above 20 ng/mL are correlated with a 122% increased risk of Alzheimer’s disease [23]. Serum 25(OH)D concentrations of below 12 ng/mL vs. above 20 ng/mL are correlated with a 43% increased risk of multiple sclerosis in women [24]. Serum 25(OH)D concentrations of less than 12 ng/mL vs. more than 12 ng/mL in early pregnancy are correlated with a 90% increased risk of multiple sclerosis in offspring [25]. Serum 25(OH)D concentrations in neonates and pregnant mothers at mid-gestation of less than 10 ng/mL vs. more than 20 ng/mL are correlated with a 142% increased risk of autism in offspring [26]. Lower vs. higher sun exposure in pregnancy is correlated with a 67% increased risk of type 1 diabetes in offspring [27]. Serum 25(OH)D concentrations of less than 12 ng/mL vs. more than 12 ng/mL in women who had a genetic risk for age-related macular degeneration (AMD) are correlated with a 6.7-fold increase in the risk of developing AMD [28]. Inadequate sun exposure has also been tied to increased risk of myopia [29], psoriasis [30], non-alcoholic fatty liver disease [31], eczema in infants [32], and cognitive problems [33]. A more detailed description of these and other similar studies are to be found in Hoel et al., 2016 [34]. The problem of inadequate sun exposure in the United States is more severe for dark skinned individuals than for those with light skin. Identified mediators produced by sun exposure include vitamin D, nitric oxide, dopamine, beta-endorphin, urocanic acid, and glutamate. Vitamin D supplementation is not an adequate substitute for sun exposure [35].

The incidence of melanoma in the United States has steadily increased at an annual rate of 3–4% from 1 case per 100,000 in 1935, when accurate records were established, to 25.8 cases per 100,000 in 2015. According to the Centers for Disease Control and Prevention (CDC), the prevalence of sunburns increased from 32% of all white adults in 1999 to 34% in 2004 [36] and up to 50% in 2012 [37]. Among white adolescents aged 12–18 in 1999, 83% reported at least one sunburn in the previous summer and 36% reported three or more sunburns in the previous summer [38]. Public health officials, such as the U.S.’s CDC, advise against “overexposure” when they should be specifically advising against sunburns. This combined with dubiously advising against moderate sun exposure and the incorrect assertion that tanned skin provides insignificant protection against sunburn may have contributed to this increase in melanoma incidence. 

While there is still vigorous debate within the scientific community regarding the desirable level of serum 25(OH)D, many scientists believe, and the scientific studies discussed above indicate, that it is between 30 and 60 ng/mL (75–150 nmol/L). A well-controlled study with simulated solar UV led to the estimate that about a half hour of midday summer sun three times a week in summer clothing would be enough for 90% of white Caucasians to achieve a serum 25(OH)D level equal to or greater than 20 ng/mL (50 nmol/L) at latitudes 30 to 55 degrees [39]. More exposure would be needed to achieve desirable levels of 30–60 ng/mL (75–150 nmol/L), and more exposure would be required in other seasons, at earlier or later times of the day, at higher latitudes, and for persons with darker skin. In light of all the variables involved and the fact that each person is different, the recommended course of action is for each person to have periodic serum 25(OH)D tests and to adjust sun exposure as necessary to reach desired levels of 25(OH)D while taking great care to avoid sunburns. If all sunburns are avoided, these levels can probably be achieved without any appreciable increase in the risk of melanoma and without significant increase in the risk of SCC or BCC. Data from the National Health and Nutrition Examination Survey (NHANES) show that 77% of Americans had serum 25(OH)D concentrations of less than 30 ng/mL in 2001–2004, up from 55% in 1988–1994 [40]. It is noted that the comprehensive 2010 vitamin D study [35] by the Institute of Medicine (IOM; now named the National Academy of Medicine) is often misinterpreted as standing for the proposition that 20 ng/mL is an adequate level of serum 25(OH)D. The purpose of the IOM study was, as the title indicates, to determine the appropriate recommendation for the dietary reference intake (DRI) of vitamin D supplements. The study correctly concluded that vitamin D supplements had not been shown to have any health benefits beyond skeletal health, and that for purposes of skeletal health a serum 25(OH)D level of 20 ng/mL was sufficient [35].

## 2. Conclusions

The public health directive regarding sun exposure and human health should be adjusted to reflect current scientific knowledge. We recommend a public health directive as follows:

All persons in the world regardless of skin color or latitude of residence, other than those with extraordinary sensitivity to sunlight, should get enough sun exposure to maintain a serum 25(OH)D level well over 20 ng/mL (desirably at 30–60 ng/mL) while taking care to avoid sunburn.

As with all things in life, we must maintain a good balance, in this case between beneficial and adverse health effects from sunlight. Besides being impractical, completely avoiding sun exposure appears to be unhealthy in many ways. The responsibility for the proper health directive within the U.S. appears to lie with the Centers for Disease Control and Prevention (CDC), and within the CDC with the National Center for Chronic Disease Prevention and Health Promotion. Responsible agencies in Europe and the rest of the world include the World Health Organization, Cancer Research U.K., the French Ministry of Social Affairs and Health, the Ministry of Health and Cancer Society of New Zealand, and Cancer Council Australia.
